# Comment on “Video-Assisted Thoracic Surgery for Tubercular Spondylitis”

**DOI:** 10.1155/2014/209248

**Published:** 2014-12-07

**Authors:** Safak Ekinci, Serkan Bilgic, Kenan Koca, Mehmet Agilli, Omer Ersen

**Affiliations:** ^1^Department of Orthopaedic Surgery, Agri Military Hospital, Agri, Turkey; ^2^Department of Orthopaedic Surgery, Haydarpasa Gulhane Military Hospital, Istanbul, Turkey; ^3^Department of Orthopaedic Surgery, Gulhane Military Hospital, Ankara, Turkey; ^4^Department of Biochemistry, Agri Military Hospital, Agri, Turkey; ^5^Department of Orthopaedic Surgery, Erzurum Military Hospital, Erzurum, Turkey

We have read the published paper by Singh et al. [1] with great interest. In their study, the authors evaluated the outcome of video-assisted thoracic surgery (VATS) in 9 patients (males = 6, females = 3) with clinicoradiological diagnosis of tubercular spondylitis of the dorsal spine. But they said “We performed video-assisted thoracoscopic surgery in 9 patients (males = 6,* females = 7*) with tubercular spondylitis of the dorsal spine at our centre from January 2009 to December 2011” in Patients and Method section.

Besides this little mistake, they said “Absolute indications for surgery in patients with spinal tuberculosis under active treatment are approximately 6% in those without neurologic deficit and approximately 60% in those with neurologic deficit” in Discussion section [1]. Although the objective of the study is noteworthy, we would like to offer additional points that should be discussed with patients with spinal tuberculosis during the decision-making and therapy planning processes.

Some scientists reported that paradoxal responses are defined as worsening of existing symptoms or the appearance of new lesions in patients who initially responded well to antituberculous therapy [2, 3].

Triple-drug antituberculous chemotherapy can play a main role in treating tuberculosis [4] if the lesion is without complications and is limited to the vertebrae. However, with proper indications, surgical procedures are superior in the prevention of neurological deterioration, maintenance of stability, and early recovery [5, 6].

Currently, there are few widely accepted classification systems based on objective data that can provide guidance on selecting the proper treatment approach for patients with spinal tuberculosis. In 2008, Oguz et al. [7] developed a classification system for spinal tuberculosis based on seven clinical and radiological criteria (abscess formation, vertebral collapse, disc degeneration, sagittal index, kyphosis, instability, and neurological problems). At this system, spinal tuberculosis is divided into three types (Type I A/B, Type II, and Type III) by using these criteria and it also recommends specific therapeutic techniques for each type. Contrary to authors, they recommended surgery for Type I B (abscess formation, one- or two-level disc degeneration, no collapse, and no neurologic deficit), Type II, and Type III patients with or without neurological deficit [7].

We believe that this classification system should be considered as a practical guide for spinal tuberculosis treatment planning in all countries.
